# KCNQ1 rs2237895 polymorphism is associated with Gestational Diabetes in Pakistani Women

**DOI:** 10.12669/pjms.326.11052

**Published:** 2016

**Authors:** Syeda Sadia Fatima, Bushra Chaudhry, Taseer Ahmed Khan, Saad Farooq

**Affiliations:** 1 Syeda Sadia Fatima, MBBS, MPhil, PhD, Department of Biological and Biomedical Sciences, Aga Khan University, Karachi, Pakistani; 2 Bushra Chaudhry, PhD, Department of Biological and Biomedical Sciences, Aga Khan University, Karachi, Pakistani; 3 Taseer Ahmed Khan, PhD. Department of Physiology, University of Karachi, Karachi, Pakistan; 4 Saad Farooq, MBBS Year 5 Student, Medical College, Pakistan

**Keywords:** Gestational Diabetes Mellitus, KCNQ1, SNP, GWAS, Body mass index

## Abstract

**Background and Objective:**

Genetic studies on gestational diabetes (GDM) are relatively scarce; moreover, limited data is available for KCNQ1 polymorphism in Pakistani pregnant women. We aimed to determine the frequency of KCNQ1 rs2237895 in GDM and normal pregnant controls and its association with GDM-related phenotypes.

**Methods:**

A total of 637 pregnant females (429 controls and 208 cases) in their second trimester were classified according to the International Association of the Diabetes and Pregnancy Study criteria in this study. Their blood samples were genotyped for KCNQ1 SNP rs2237895 using PCR-RFLP method and sequencing. Fasting and two hour-post glucose load blood levels, serum HbA1c, insulin, and anthropometric assessment was performed.: Pearson’s Chi Square test, Mann- Whitney U test, and regression analyses were performed. A p-value of <0.05 was considered significant.

**Results:**

The variant genotyped was in Hardy-Weinberg equilibrium (p>0.05). The rs2237895 showed an association with GDM (OR 2.281; 1.388-3.746: p <0.001) and remained significant after multiple adjustments for age and body mass index (OR 2.068; 1.430-2.997: p=0.005). The C allele showed positive association with insulin level, and HOMA-IR in study subjects.

**Conclusions:**

This study identifies that KCNQ1 rs2237895 polymorphisms might be associated with risk of GDM in Pakistani population and that it is related to higher glucose levels and insulin resistance. Further large scale studies are required to consolidate on the functional aspect of this polymorphism.

## INTRODUCTION

Gestational diabetes mellitus (GDM) is defined as glucose intolerance that first occurs or is first identified during pregnancy.^1^ Although the true prevalence of GDM is unknown, a 16–27% prevalence is reported worldwide,^2^ whereas, 8% pregnant females suffer from GDM in Pakistan.^3^-^5^ GDM not only increases the likelihood of several maternal and perinatal complications including delivery by cesarean section, neonatal hypoglycemia and adiposity, but women with GDM and their offspring are also at an increased risk of developing diabetes later in life.^5^ GDM has many etiological factors and till date a particular pathophysiological mechanism singularly responsible is unidentified. The genome wide association studies have identified various susceptibility genes, including several type 2 diabetes mellitus (T2DM) such as PPARG, TCF7L2, FTO, CDKN2A/2B, and IRS1 etc. to be associated with the risk of T2DM, though their definite relation with GDM remains to be explored further.^6^,^7^

Recently, KCNQ1 (potassium voltage gated channel, KQT-like subfamily, member1) was added to the candidate genes conferring susceptibility to T2DM.^8^ KCNQ1 is located at chromosome 11p15.5, and encodes the pore-forming potassium (K+) channel alpha-subunit. It is expressed in the heart, inner ear, stomach, small and large intestine, liver, kidney, adipose tissue and the pancreas. So far its role is well researched in the pathogenesis of long QT syndrome and atrial fibrillation,^9^ where it is responsible for encoding a voltage-gated potassium channel required for the repolarization phase of the cardiac action potential.^10^

Insulin release from beta cells (ß) of pancreases is controlled by intricate interaction between ATP-sensitive potassium (KATP) channels, voltage-dependent potassium (Kv) channels and voltage-dependent calcium channels.^11^,^12^ KCNQ1 is responsible for membrane repolarization of pancreatic ß cell, leading to cessation of calcium influx and subsequently insulin secretion. Mutations in this channel may be responsible for premature termination of calcium influx which leads to decreased insulin secretion in patients with T2DM and GDM.^11^,^12^

A strong association between KCNQ1 polymorphisms and T2DM is reported both in the East Asian and European populations.^13^,^14^ The single-nucleotide polymorphisms (SNPs) reported has the second largest effect size next to TCF7L2. The following SNP (rs2237897, rs2283228, rs2237895, rs2074196 and rs2237892) of this gene have recently been shown to confer susceptibility to T2DM. In addition, the risk allele of rs2237892 was associated with impaired insulin secretion, which may be intermediated through an effect on beta cell function,^15^,^16^ whereas the C alleles of rs2237892 and rs2237895 were found to be associated with increased risk of GDM in Korean population.^17^

Besides these favorable results, uncertainty exists about their association with GDM or its related phenotypes. Thus, we aimed to investigate the association of common genetic polymorphism of KCNQ1 rs2237895 in gestational diabetic and normal pregnant controls. In addition, this SNP was examined for its possible association with GDM-related clinical phenotypes.

## METHODS

Late second trimester pregnant women from the antenatal care clinics of Aga Khan University and Abassi Shaheed Hospital in Karachi, Pakistan were enrolled in this case-control study. The investigation was carried out between February 2014 and February 2016. The institutional ethics committee approved the research protocol; written informed consent was obtained from all participants.

A total of 637 pregnant females were recruited by employing convenience random sampling technique for this case-control study. This sample size was considered sufficient to achieve 80% power and detect an odds ratio of at least 2 among GDM women. The two sided level of significance was set at 5% as calculated by NCSS/PASS version 11 software for power analysis and sample size.^18^ The study group included 208 pregnant women diagnosed with gestational diabetes (GDM case) according to the Glucose Tolerance Test (GTT) criteria proposed by the International Association of Diabetes and Pregnancy Study Group (IADPSG) i.e. a fasting glucose ≥92 mg/dL and/or 1 h: ≥180 mg/dL (10.0 mmol/L) and/or 2 h: ≥153 mg/dL (8.5 mmol/L) (when any of the following plasma glucose values are exceeded, the subject is considered as having GDM). The control group included 429 normo-glycemic pregnant women, with blood glucose levels below the above mentioned criteria. All study subjects (whether case or control) were excluded if they had diabetes before pregnancy or developed diabetes before 24 weeks of gestation, impaired fasting glucose or impaired glucose tolerance, history of hypertension, twin pregnancies, taking hormonal support or any condition affecting the maternal fetal health.

The weight at booking and anthropometric measurements of all participants was recorded from the patient record cards, while current weight of subjects was measured in kilogram on a digital weighing scale in light clothing without shoes (with an accuracy of ±100g) while body mass index (BMI) was calculated.

Ten ml of venous blood was collected. Serum was obtained after centrifugation of blood samples and was immediately frozen at -80 C till further analyzed. Serum Insulin was measured using commercially available ELISA assay (cat# KAP1251, DIA source Immuno Assay S.A., Belgium), while insulin resistance (HOMA-IR= Glucose (mg/dl) X Insulin (µIU/ml)/ 405) and insulin sensitivity (QUICKI =1/Log (fasting insulin μIU/ml) + log (fasting glucose mg/dl) were calculated.

DNA was extracted from whole blood as per the vendor’s instruction by commercially available Qiagen DNA extraction kit (Cat. number 51185, Valencia, CA, USA). The quantification of extracted DNA was performed by measuring the UV absorbance of the samples using a Nanodrop-ND1000 (Thermo Fisher Scientific, Waltham, MA). A ratio of ~1.8 was considered acceptable for confirming the purity of extracted DNA.

Primers were designed using NCBI primer designing tool for KCNQ1 rs2237895 (Forward CTCAGCCTGCAGTGTCCAGG; Reverse AAGGGACAGAGCTGCTCCCAA). Polymerase chain reaction (PCR) was performed using the Ruby Taq PCR Master mix 2X (cat# 71191, Affymetrix, USA) as per the manufacturer’s instructions. The amplicons were digested with restriction enzyme SmaI (Cat #R0141L, New England Biolabs, USA) according to the standard instructions of the manufacturer. The digested fragments were electrophoresed in 2% Agarose gel.

**Fig.1:**
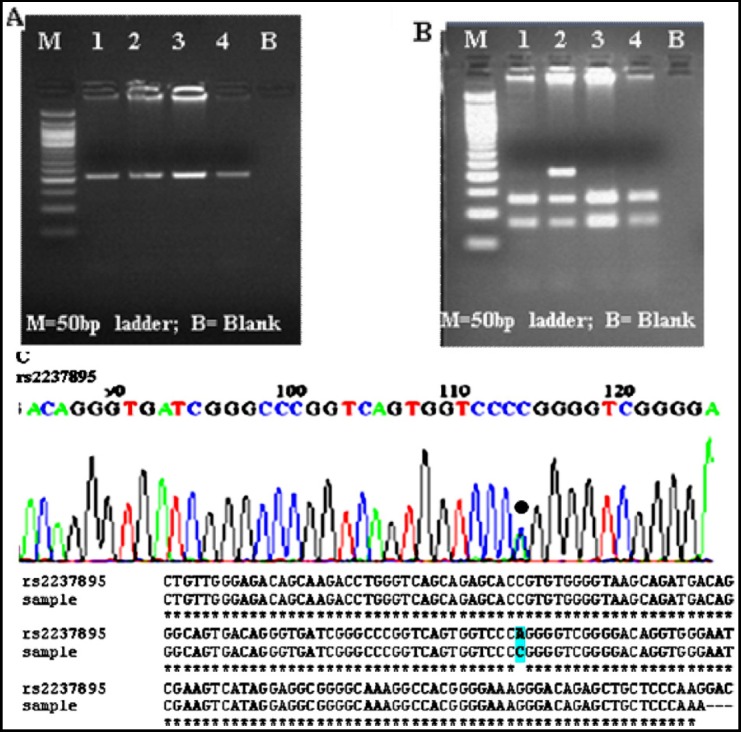
(A-C): Gel Electrophoresis and Sequencing Chromatogram of KCNQ1 SNPs. Where M is the 50bp DNA marker and B are negative controls in the PCR reaction. A: PCR amplification shown as bands on 2% agarose gel for rs2237895 (218bp) samples numbered 1-4. B: RFLP fragment [3 homozygous mutated: 140bp/78bp (C/C) for samples number 1, 3 & 4; 1 heterozygous carrier: 218bp/140bp/78bp (A/C)] for sample numbered 2 respectively. C: Sequencing Chromatograms showing the mutations as dot and highlighted section in the aligned sequence.

Genotypes were scored by an independent person, who was blinded about the case control status of the study subjects. In the additive model, genotype of non-risk allele homozygote (C/C), heterozygote (A/C) and risk allele homozygote (A/A) were coded as 0, 1 and 2 respectively. Further, negative controls were placed in duplicates in each run, to ensure correct genotyping. Overall call rate for genotype data was >97%. A genotyping quality control was performed in 50% of the samples by duplicate checking (rate of concordance in duplicates was >99%). Further reconfirmations of genotyping accuracy were made by sequencing 20% of the samples with detected polymorphic variants (discrepancy rate was <0.20%) (ABI3730XL, automated DNA sequencer, Macrogen, Korea) (Fig.1).

### Statistical Analysis

Statistical analyses were conducted using the IBM Statistical Package for the Social Sciences (IBM SPSS version 21; IBM Corp Inc, Armonk, NY). Descriptive analysis of categorical data was presented in terms of frequencies and percentages whereas that for continuous variables was expressed as mean ± standard deviation. Mann Whitney U test and Pearson chi-square test of independence were used for comparing continuous and categorical variables wherever applicable. Hardy–Weinberg equilibrium (HWE) was calculated for SNP data. SNP data was analysed for genotype and allele frequency determination by applying chi-squared statistics. Significance of risk allele with study parameters was determined with regression analyses. Genotypes were allotted code of 0/1/2 rendering the number of minor alleles under an additive model of inheritance.

## RESULTS

Six hundred and thirty-seven pregnant females were recruited in this study. The GDM group had a higher BMI, pre and post load GTT, HBA1c and insulin levels (p<0.01) when compared to pregnant controls (Table-I).

**Table-I:** Clinical and biochemical data of cases and controls.

Variables	Control n=429	Case n=208

	Mean ± S.D	Mean ± S.D
Age (in year)	25.97 ± 4.87	27.31± 5.56
Weight at Booking (kg)	57.55 ± 10.15	63.84± 12.58**
BMI at Booking (kg/m^2^)	22.77 ± 4.19	24.83 ± 5.13**
GTT 0 hr (mg/dl)	78.69 ± 8.96	106.23 ± 20.70 **
GTT 2 hr (mg/dl)	124.22 ± 11.94	177.33 ± 45.57**
HbA1c (%)	N.A	5.79 ± 1.43
Insulin (IU/mL)	13.28 ± 5.26	37.08 ± 7.66**

BMI (body mass index), GTT (glucose tolerance test). Values expressed as Mean ± SD. Groups compared by Mann Whitney U test for continuous data.

** p<0.01

Genotype and allele frequencies of the study subjects are given in Table-II. The genotyped variant showed a strong association with GDM (p<0.001). The rs2237895 C allele depicted a significant risk association with GDM (p<0.001).

**Table-II:** Genotypes and Allele Frequencies of KCNQ1 rs2237895 polymorphism.

Genotype	Control (n=429)	Case (n=208)	P value	Allele	Group	Allele Data		

	rs2237895					Frequency n (%)	OR (95%CI)	P value
AA	175 (40.8)	51 (24.5)	<0.001	C	Control	320 (37.50)	1.557 (1.228-1.973)	< 0.001
AC	188 (43.8)	114 (54.8)			Case	216 (51.92)		
CC	66 (15.4)	43 (20.7)		A	Control	538 (62.50)		
					Case	200 (48.08)		

***Genotype AA:*** homozygous dominant; **AC:** heterozygous mutated; **CC:** homozygous mutated. A is the major allele and C is the minor allele. Frequencies are given as absolute values with percentage given in parenthesis. The Hardy Weinberg Equilibrium (HWE) stats for the study cohort was > 0.05 P value <0.05 calculated by Pearson’s x2 square test.

Further, regression analysis was performed on unadjusted genotypes and after adjusting with risk factors such as age and pre pregnancy BMI. A significant association was observed with GDM (OR 2.281; p = 0.0002), which remained independently linked with GDM risk (OR 2.068; p=0.005) even after multiple comparison under additive model (Table-III).

**Table-III:** Association of KCNQ1 SNP with GDM assessed under Additive model.

Unadjusted Odds Ratio			Adjusted for Age and BMI	

Genotype	Odds Ratios	95.0% C.I Odds Ratio	Odds Ratios	95.0% C.I Odds Ratio
rs2237895	2.281**	1.388-3.746	2.068*	1.430-2.991

* OR with p<0.05 considered as significant;

** OR with p<0.001.

We also investigated the association of KCNQ1 variant with quantitative traits associated with obesity and glucose homeostasis in the study cohort. Linear regression analysis revealed a moderately significant association of ‘C’ allele with insulin levels (β = -0.238 p = 0.0007), HbA1c (β = -0.123 p< 0.001) while weak association were seen with HOMA-IR (β = -0.021 p< 0.001) and fasting blood glucose (β = -0.043 p< 0.001).

## DISCUSSION

There are many proposed theories such as polymorphisms in PPAR2, IGF2BP2, CDKL1 and CDKN2a in the quest to find a link between genetic factors and GDM.^7^ Many studies have linked T2DM with polymorphisms in KCNQ1 gene.^16^,^11^,^13^,^19^ but few with GDM. We focused on rs2237895 SNP in the KCNQ1 gene which is known to be a susceptible T2DM candidate gene, and it showed a possible sound association with GDM in our study (Table II and III). Our findings are in line with the initial data on the link between KCNQ1 and GDM which came through studies done in Korean population. It was reported that the C allele of rs2237895 was associated with increased risk of GDM (OR=1.24; p=0.005).^6^,^20^,^21^ Similar results to our study were found in a Chinese population.^22^ We also report a risk allele frequency of 52% in GDM positive females, which is somewhat close to the risk allele frequency of 45% reported in Indian Punjabis with T2DM,^13^ 35% in Chinese females with GDM,^22^ 33% in Korean GDM females^20^ and 34.5% in Chinese subjects with T2DM.^23^

Similarly, the minor allele frequency reported in our study was 37.5% in our control population (Table-II), which is close to a previously published result of 42%.^13^ The difference in frequencies reported could be due to a different set of ethnic population at a different geographic location tested. A moderately significant association of ‘C’ allele with insulin levels, HbA1c, insulin resistance and fasting glucose levels was observed in our study similar to a previous report.^13^ Additionally, in two recent related studies for Pakistani population it was reported that SNPs in or near PPARG, TCF7L2, FTO, CDKN2A/2B, and KCNQ1 may have potential associations with T2DM, with similar effect sizes to those seen in European populations.^6^,^24^ Though, this is an exciting finding further research is required to establish an affirmative causal link.

We also observed that both weight and body mass index (BMI) values were higher in our GDM cases in comparison to controls (Table-I). Asian’s are known to have a higher body fat proportion even at a lower BMI value in comparison to Caucasian’s,^26^ and this situation might lead to early development of adverse effects during pregnancy. Though, we failed to identify any significant association in terms of weight and BMI with the minor allele in our study population. Despite the fact that weight of the baby is controlled by the anthropometric measures of the parents’ via genetic bond, mother’s weight has a supplementary effect, controlling the intrauterine environment. This suggests that KCNQ1 polymorphism may not have a direct impact on the weight status of an individual but act by other pathways to induce insulin resistance in GDM and/or T2DM.

The associations between KCNQ1 polymorphisms and increased risk for GDM not only indicate similar pathogenic mechanisms with T2DM but also make KCNQ1 a novel potential therapeutic target for both T2DM and GDM. Our study was limited in a way that we were unable to recruit large number of study participants due to cultural constraints about genetic studies. More so, we did not focus on the lifestyle factors or environmental factors, such as diet and physical activities, which prevents us from analysing any interaction between rs2237895 and other factors on GDM. Nevertheless, the rs2237895 polymorphism was in HWE in the both groups, suggesting the randomness of subject selection which is a strength of our study.

## CONCLUSION

This study identifies that KCNQ1 rs2237895 polymorphisms might be associated with risk of GDM in Pakistani population and that it is related to the higher glucose levels and insulin resistance. Further large scale studies are required to consolidate on the functional aspect of this polymorphism.
